# Supplementary material for: miRNA- and Cell Line-Specific Constraints on Precursor miRNA Processing of Stably Transfected Pancreatic Cancer and Other Mammalian Cells
Source: Int J Mol Sci. 2024 May 23;25(11):5666. doi: 10.3390/ijms25115666 (PMC11172344; doi:10.3390/ijms25115666)

Supplementary data

Supplementary Table S1 miRNA sequences used in stable vector design

D**esign 1**: **~ 70 base pair flanking region (native pri-miRNA sequence)**
**>hsa-miR-29b-1**
TTTCCTTTCTAGGTTGTCTTGGGTTTATTGTAAGAGAGCATTATGAAGAAAAAAATAGATCATAA<br>AGCTTCTTCAGGAAGCTGGTTTCATATGGTGGTTTAGATTTAAATAGTGATTGTCTAGCACCAT<br>TTGAAATCAGTGTTCTTGGGGGAGACCAGCTGCGCTGCACTACCAACAGCAAAAGAAGTGAAT<br>GGGACAGCTCTGAAGTATTTGAAAGCAAC
**>hsa-miR-378e**
GGAAGTAAGCACATTGTTCAAGGAAACTAGGACGAGCTAGTGGGGTGGACAAGCTGCATCCT<br>GAACCTGGCTCTGCCTGACTCCAGTGTCCAGGCCAGGGGCAGACAGTGGACAGAGAACAGT<br>GCCCAAGACCACTGGACTTGGAGTCAGGACATCATTTTAACAGCTTTATCGAGATGTAATTTAC<br>ATGCCATACAATTTACCCAAAGTGTACAATCATTGACT
**>hsa-miR-1290**
TTGGAGAATTTGCCGAACAAGTTGAGCATCCCTACTCCCAAGTTCCAAAGTCCAAAATGCTCC<br>AATGAGCATTTCCTTTGAGCGTCACGTTGACACTCAAAAAGTTTCAGATTTTGGAACATTTCGG<br>ATTTTGGATTTTTGGATCAGGGATGCTCAACTTGTCATGCGTTCCTCATACGCTTATGTGGTGG<br>ATTCAGTGAGATGATCCTGTCTCACTTAGGTTAATG
**>hsa-miR-2467**
ACTTAGCCATCGACTTCTGTCTTGCGTCCACATGAGAGAGAAGACGGTGGCCACTGGACAGA<br>CGCTTGGACAGGCACCTGAGGCTCTGTTAGCCTTGGCTCTGGGTCCTGCTCCTTAGAGCAGA<br>GGCAGAGAGGCTCAGGGTCTGTCTGGGTCACTCTCTTTGAGATTGCCGGAACGCTTACACAC<br>ATCTTTCCCTGGGTGGAATCGTCTTTGTCTGAA
**>hsa-miR-6831**
TGTACCCAGCGGTAGCACCCAGTGGCAGCGCCCAACCTGGGAACTAGGAGATGCAGAGGAC<br>CCAGGCACGGTAGGTAGAGTGTGAGGAGGAGGTCTGAGCCCATGTGTGGACCTAGGTCTGC<br>TGTTAAACTGACTAACTCCCACTCTACAGGGAACGGAGGGGATCTGGGGACTGCGGCCCCCC<br>AAAGGGAGATCCTTCTCCAGCCTGGAGAGTTCACTGGA
**>hsa-miR-708**
CTGACTTCTCCTGCCCCAGGGGTTTTCAGAAACCTAACCCCCATGGTTGGCGAGGGACTGCT<br>GTGTGTGAAATGGTAACTGCCCTCAAGGAGCTTACAATCTAGCTGGGGGTAAATGACTTGCAC<br>ATGAACACAACTAGACTGTGAGCTTCTAGAGGGCAGGGACCTTACCCTAGTCATCTCTCTTCT<br>CACCCTGCACACCCTCCCTGAGGGATCTCATCCATCCCCATGGCGTCAACCACCACCTGAGG<br>A
**Design 2 – 76 base pair flanking region from miR-708**
**e.g. miR-708 FR + miR-1290 pre-miRNA sequence**
CTGACTTCTCCTGCCCCAGGGGTTTTCAGAAACCTAACCCCCATGGTTGGCGAGGGACTGCT<br>GTGTGTGAAATGGTGAGCGTCACGTTGACACTCAAAAAGTTTCAGATTTTGGAACATTTCGGAT<br>TTTGGATTTTTGGATCAGGGATGCTCAACCTTACCCTAGTCATCTCTCTTCTCACCCTGCACAC<br>CCTCCCTGAGGGATCTCATCCATCCCCATGGCGTCAACCACCACCTGAGGA

Supplementary Table S2 Fold change values from microarray for miRNA panel in normal versus tumour versus F1 samples.

miRNA	T vs N FC	P value	T vs F1 FC	P value
**miR-29b**	2.52	0.23	5.82	0.02
**miR-1290**	1.39	0.32	15.40	0.006
**miR-2467**	1.48	0.17	7.91	0.00000002
**miR-6831**	4.82	0.02	1.81	0.01
**miR-708**	7.6	0.0085	-25.34	0.0019

Supplementary Table S3 Parental miRNA expression level profiling. Data presented as Ct average of biological triplicates.

miRNA	Mia PaCa-2	PANC-1	HEK293T	SK-OV-3	CHO-K1
**miR-29b**	29.8	30.3	Undetected	35.5	Undetected
**miR-1290**	Undetected	33.2	29.7	Undetected	Undetected
**miR-2467**	Undetected	Undetected	Undetected	Undetected	Undetected
**miR-6831**	Undetected	Undetected	Undetected	Undetected	Undetected
**miR-708**	Undetected	35.2	30.2	Undetected	Undetected

Supplementary Table S4 Minimum Free Energy (MFE) values for design 1 (genomic flanking regions) and design 2 (miR-708 flanking regions) as predicted by the RNA folding algorithm, Vienna RNA fold.

miRNA	Design 1 MFE (Kcal/mol)	Design 2 MFE (Kcal/mol)
miR-29b-1-5p	-60.4	-87.1
miR-1290	-95	-66.2
miR-2467-3p	-91.5	-95.9
miR-6831-5p	-79.6	-70.4
miR-708-5p	-92.5	-

Supplementary Figure S1 Transfection efficiency determined by GFP expression via microscopy in Mia PaCa-2 (top panel) and toxicity assay with serial dilutions of doxycycline concentrations from 100 ng to 1 ng in Mia PaCa-2 and PANC-1 cells (bottom panel).

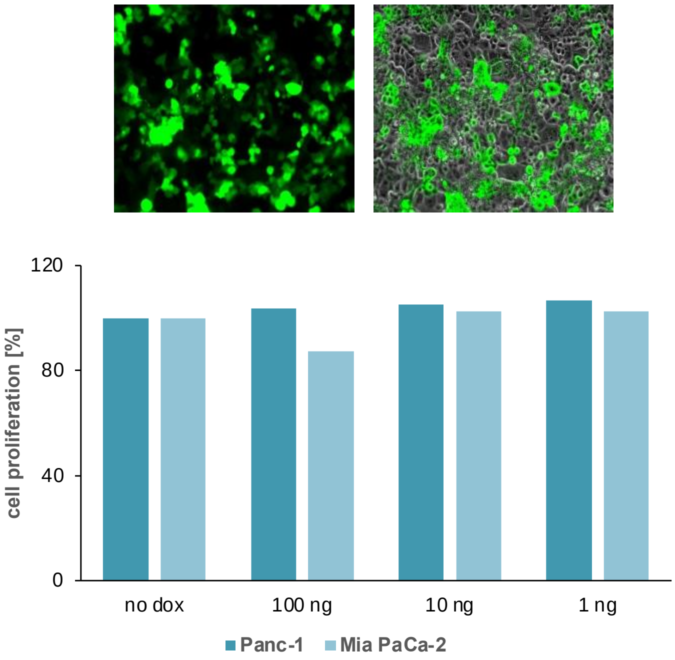

Supplement: Supplementary file 1 [file ijms-25-05666-s001.zip › ijms-2981386-supplementary.pdf]
